# The Coordination Behavior of Two New Complexes, [(C_7_H_10_NO_2_)CdCl_3_]_n_(I) and [(C_7_H_9_NO_2_)CuCl_2_] (II), Based on 2,6-Dimethanolpyridine; Elaboration of the Structure and Hirshfeld Surface, Optical, Spectroscopic and Thermal Analysis

**DOI:** 10.3390/ma15051624

**Published:** 2022-02-22

**Authors:** Sabrine Hermi, Abdullah A. Alotaibi, Abdullah M. Alswieleh, Khalid M. Alotaibi, M. G. Althobaiti, Christian Jelsch, Emmanuel Wenger, Cherif Ben Nasr, Mohamed Habib Mrad

**Affiliations:** 1 Materials Chemistry Laboratory, Faculty of Sciences of Bizerte, University of Carthage, Zarzouna 7021, Tunisia; sabrin94hermi@gmail.com (S.H.); cherif_bennasr@yahoo.fr (C.B.N.); 2 Department of Chemistry, College of Sciences and Humanities, Shaqra University, Ad-Dawadmi 11911, Saudi Arabia; aaalotaibi@su.edu.sa; 3 Department of Chemistry, College of Science, King Saud University, Riyadh 11451, Saudi Arabia; aswieleh@ksu.edu.sa (A.M.A.); khalid.m@ksu.edu.sa (K.M.A.); 4 Department of Physics, College of Science, Taif University, P.O. Box 11099, Taif 21944, Saudi Arabia; m.althobaiti@tu.edu.sa; 5 CRM2, CNRS, Université de Lorraine, 54000 Nancy, France; christian.jelsch@univ-lorraine.fr (C.J.); emmanuel.wenger@univ-lorraine.fr (E.W.)

**Keywords:** coordination compound, X-ray structure, Hirshfeld surface study, IR spectroscopy, UV-visible absorption, TG-DTA

## Abstract

Two novel complexes, [(C_7_H_10_NO_2_)CdCl_3_]_n_(I) and [(C_7_H_9_NO_2_)CuCl_2_],havebeen synthesized and characterized. Single crystal X-ray diffraction revealed that in compound (I), 2,6-dimethanol pyridinium acts as a monodentate ligand through the O atom of the hydroxyl group. Contrarily, the 2,6-dimethanol pyridine ligand interacts tridentately with the Cu(II) ion via the nitrogen atoms and the two oxygen (O, O’) atoms of the two hydroxyl groups. The structure’s intermolecular interactions were studied using contact enrichment ratios and Hirshfeld surfaces. Following metal coordination, numerous hydrogen connections between entities and parallel displacement stacking interactions between pyridine rings dictate the crystal packing of both compounds. The aromatic cycles generate layers in the crystal for both substances. Powder XRD measurements confirmed the crystalline sample phase purity. SEM confirmed the surface homogeneity, whereas EDX semi-quantitative analysis corroborated the composition. IR spectroscopy identified vibrational absorption bands, while optical UV-visible absorption spectroscopy investigated optical properties. The thermal stability of the two materials was tested using TG-DTA.

## 1. Introduction

Coordination compounds based on aromatic acids have sparked huge interest due to their fascinating geometrical properties as well as their intriguing applications in a variety of fields, such as the fields of catalysis, gas storage, and biochemistry. Additionally, this family of materials has contributed significantly to our knowledge of low-dimensional magnetic systems and semiconductor materials [1,2,3,4,5,6,7,8,9,10,11].

The choice of the metal and the ligand is critical in generating the desired compound with developed physicochemical characteristics. In this regard, compounds based on cadmium (II) and copper (II) offer a potential class of materials with different structural patterns [12,13,14,15,16,17,18,19,20,21,22]. Chemists are interested in cadmium-based hybrid complexes because of their structural flexibility, which is connected to the variety of coordination numbers and geometries of the Cd cation (with the d^10^ configuration) relying on crystalline packing and ligands. Several anionic chlorocadmate(II) complexes have recently been studied using X-ray diffraction, and the majority of them were found to be made of polynuclear or mononuclear anions, with the anionic sub-network of the crystal consisting of a simple discrete octahedron and a simple tetrahedron [23,24,25,26].

The existence of an active Jahn–Teller effect in the electronic system d^9^ causes the diversity of the structural models of Cu (II). The hybrid compounds based on Cu^2+^ and their characteristics have a wide range of attraction in inorganic chemistry, as well as in a variety of areas spanning from solid-state physics to bio-inorganic chemistry. Due to the extreme plasticity of the coordinating sphere metal, which leads to a broad range of structures with varied coordination numbers, geometries and nuclear properties, copper halides are of considerable interest [27,28,29,30].

Among the different aromatic ligands, pyridine-2,6-dimethanol has received great interest in chemistry because of its varied chelating properties [31,32,33,34,35]. It is a flexible poly functional ligand due to the availability of multiple donor sites that may coordinate with metals in neutral, monoanionic or dianionic forms. In reality, this type of ligand has three donor sites and may operate as a tridentate chelating ligand, notably the pyridine nitrogen and the two hydroxyl oxygen atoms, which give rise to complexes of mononuclear cells. Deprotonated nitrogen atoms can coordinate with metals, whereas protonated nitrogen atoms can engage in hydrogen bonding [36].

In this paper, we deal with the crystal structures of two new complexes. The first one is based on cadmium—catena(2,6-dimethanolpyridiniumtrichlorocadmate(II) of crude formula [(C_7_H_10_NO_2_)CdCl_3_]_n_(complex(I)); and the second compound is based on copper—(2,6-dimethanolpyridine)copper(II)dichloride of the general chemical formula [(C_7_H_9_NO_2_)CuCl_2_] (complex (II)). In this work, we present the structural characteristics given by X-ray diffraction, Hirshfeld surface analysis, the vibrational modes observed byFT-IR, optical properties and also thermal properties according to the TG-DTA coupling.

## 2. Materials and Methods

### 2.1. Synthesis of Metal Complexes

To prepare catena(2,6-dimethanolpyridiniumtrichlorocadmate(II))(I) and (2,6-dimethanolpyridine)copper(II)dichloride (II), an equimolar quantity of MCl_2_(M = Cd, Cu) was mixed with a methanolic solution of 2,6-dimethanolpyridine, followed by 10 mL of concentrated hydrochloric acid. The mixture was refluxed at 60 °C for 4 h. Pure crystals of compounds (I) and (II) suitable for XRD analysis were obtained after three weeks of slow evaporation at room temperature. Merck (Darmstadt, Germany) provided all of the reagents, which were utilized without additional purification.

### 2.2. Investigation Techniques

We employed a number of approaches to investigate the two produced complexes in the remaining part of our examination. The morphology and the elemental composition were observed using a scanning electron microscope JEOL-6610-LVSEM spectrometer (JOEL–IT 300, Tokyo, Japan), operated at an acceleration voltage of 10 KV, coupled with an energy-dispersive X-ray spectrometry detector (Oxford X-Max Micro-analysis) (EDX, Oxford, UK) to obtain semi-quantitative elemental results about a specific location for both complexes (I) and (II). It is worth noting that a platinum-rich tape was used to partly cover the sample in order to minimize charge effects. Then, suitable crystals of (I) and (II) were selected with a stereo-microscope and then mounted for XRD data collection on a Rigaku OD SuperNova diffractometer (four-circle kappa geometry goniometer) (Rigaku Oxford Diffraction, SuperNova, AC3, TUD) with an Atlas CCD detector (Agilent Technologies), and a Bruker D8 Venture diffractometer with a PHOTON III 14 CPAD detector (bleuscientific, UK), respectively. Both data collections were carried out with a microfocus X-ray source at Mo(Kα) energy. CrysAlisPRO [37] and APEX4 [38] software were respectively used for (I) and (II)to optimize the data collection strategy after cell parameters and orient matrix determination and realize data processing (integration, scaling and absorption corrections by the multi-scans method).The structure was solved with the SHELXT [39] structure solution program using intrinsic phasing and refined with the SHELXL [40] refinement package using least squares minimization.Hydrogen atoms were initially located in Fourier residual maps, except in the methanol group, where they were placed in geometrically idealized positions. During the structure refinement, the HX distance of all H atoms was constrained to standard values. Crystal data and refinement details of compounds (I) and (II) are provided in Table 1, and the designated bonds and angles can be observed in Table S1 (Supplementary Materials).

For the remaining physical characterizations, single crystals were chosen based on their morphologies. The single crystals were then pulverized into a polycrystalline powder using an agate mortar. Powder X-ray diffraction (PXRD) was used to verify the powder’s purity. The PXRD was measured on a Siemens D5000 powder diffractometer (Siemens, Aubery, TX, USA) with Cu-Kα monochromatic radiation (1.542) and 2*θ* angular range from 5° to 50°.

### 2.3. Spectroscopic Measurements

The findings for the solid-state infrared (IR) spectroscopic measurements were obtained using a Fourier transform infrared spectrometer with a range spanning from 400 to 4000 cm^−1^ on a Perkin Elmer Spectra 100 spectrometer (PerkinElmer, Waltham, MA, USA). A Perkin Elmer Lambda 11UV-Vis spectrophotometer (PerkinElmer, Waltham, MA, USA) was used to obtain the UV absorption spectra of the polycrystalline powders (I) and (II).

### 2.4. Thermal Study

The thermogravimetric analysis and differential were performed in an inert environment using a TGA Q500-TA thermal analyzer in the temperature range of 280–700 K at a scanning rate of 5 K.min^−1^.

### 2.5. Computational Methods

The fingerprint plots of contacts around the organic molecule in the two crystal structures were computed with the program Crystal Explorer 17.5 [41]. The contact statistics and enrichment ratios were obtained with the program MoProViewer [42]. In that case, the Hirshfeld surface was computed around all entities (metal cation, each chloride anion and organic molecule) constituting the asymmetric unit. In all Hirshfeld surface calculations, only the main conformation of the disordered methanol group was considered. For this, moieties not in contact with each other in the crystal packing were selected to obtain an integral Hirshfeld surface around each item. The highly charged H-O/H-N atoms bound to nitrogen or oxygen were differentiated from the hydrophobic hydrogen atom H-C linked to a carbon atom.

## 3. Results and Discussion

### 3.1. Powder X-ray Diffraction Patterns and SEM/EDX Analysis

**Figure 1:**
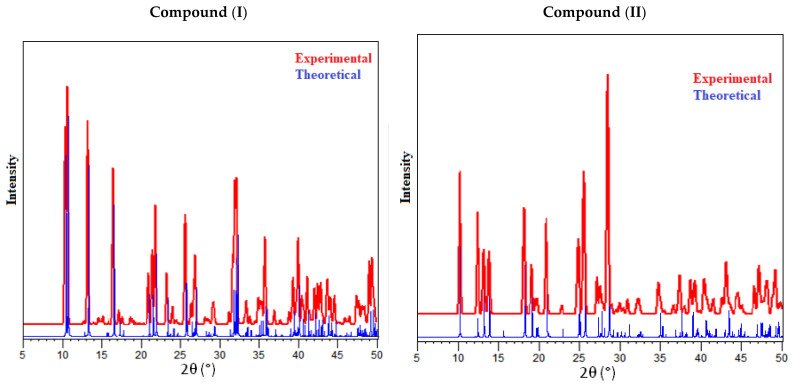
The powder diffraction patterns of compounds (I) and (II)at room temperature compared with their simulated patterns.

PXRD was carried out to confirm the phase purity of the synthesized materials. The experimental and simulated patterns of (I) and (II) seen in Figure 1 are in good agreement. It is worth mentioning that low temperature data were systematically shifted to a higher 2-theta angle due to cell contraction. However, all of the diffraction peaks matched well with the pure phase with respect to their positions. This confirms the purity and homogeneity of the synthesized materials.

**Figure 2:**
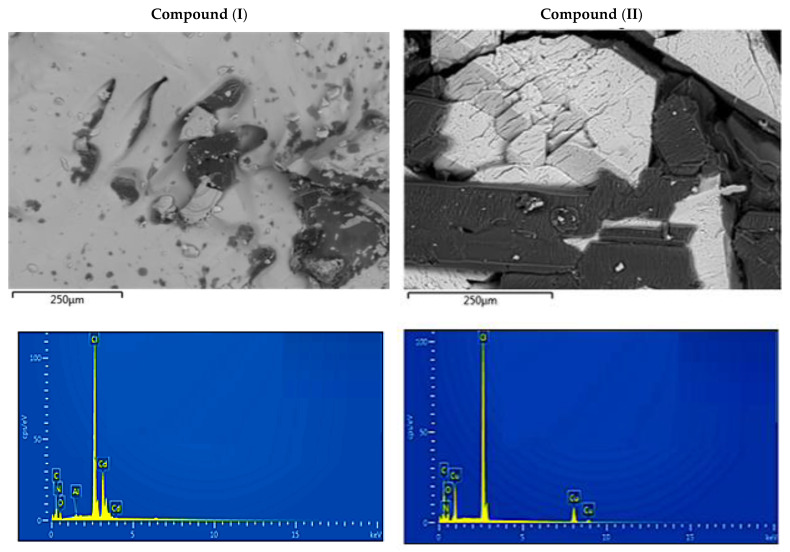
SEM micrographs with 250 µm resolution and EDX analysis of complex (I) and complex (II).

Figure 2 depicts the SEM images as well as the typical EDX spectrum. As seen by SEM micrographs, the surface of both hybrid compounds appears to be flat, indicating high crystal quality, whereas EDX examination in high contrast zones verified the existence of heavy element compositions in the crystals and showed the presence of carbon, nitrogen, oxygen, cadmium and copper signals.

### 3.2. Crystal Structure

Diverse structures have been reported for complexes of 2,6-dimethanolpyridine with the (O, N, O’) donor set. Many coordination polymers have pyridine fragments that can bind to metal atoms in different ways, like tridentate, bidentate, monodentate, or even nonchelate. The case of [C_7_H_10_NO_2_]Cl [43] and (C_14_H_20_N_2_O_4_)[SiF_6_] [44] isa typical example of a nonchelated2,6-dimethoxypyridinium ligand. In the trans-Pt-complex [45], the 2,6-dimethanolpyridine ligand acts as a monodentate ligand, whereas in the oxo-Tc (V) complex [46], this ligand is a bidentate and in pyridine-2,6-dicarboxylato)-((pyridine-2,6-diyl)dimethanol)-copper(ii) hemihydrate [47] it acts as a tridentate chelate. To better understand the ligand’s structural properties and with the aim to develop a new class of coordination polymers using 2,6-dimethanolpyridine as a coordinative building block, we present in the following section the crystal structures of catena(2,6-dimethanolpyridiniumtrichlorocadmate(II))(complex (I)) and (2,6-dimethanolpyridine)copper(II) chloride (complex(II)), where complex (I) is the first coordination compound of the ligand with cadmium to be reported, and complex(II) is another complex compound among the 21 ligand copper structures discovered in CSD.

#### 3.2.1. Crystal Structure of [(C_7_H_10_NO_2_)CdCl_3_]_n_(I)

**Figure 3:**
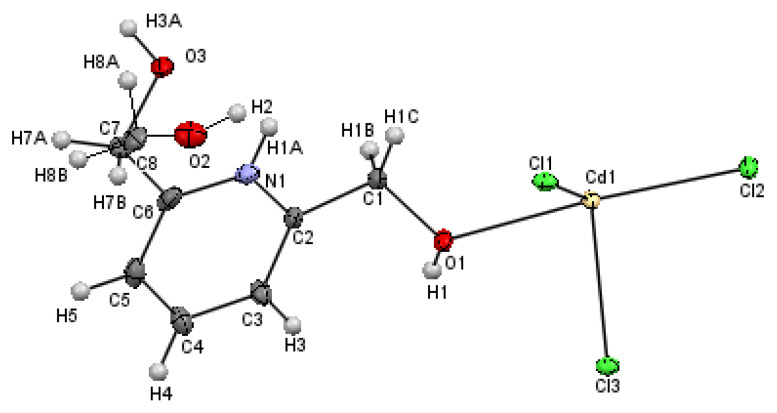
ORTEP plot showing at the 50% probability level the anisotropic displacement parameters of complex (I), with the occupancy of 0.477(2):0.523(2) for the C7/C8 and O2/O3 groups, respectively.

Figure 3 depicts the ORTEP plot with the atoms labeled, revealing that the asymmetric unit consists of one cadmium atom, Cd1, surrounded by three chlorine (Cl1, Cl2 and Cl3) atoms and one 2,6-dimethanol pyridinium linked to the metal ion via the oxygen atom O1. The ligand has a disordered form of the methanol group with the occupancy of 0.477(2):0.523(2) for the (C7, O2) and (C8, O3) groups, respectively.

**Figure 4:**
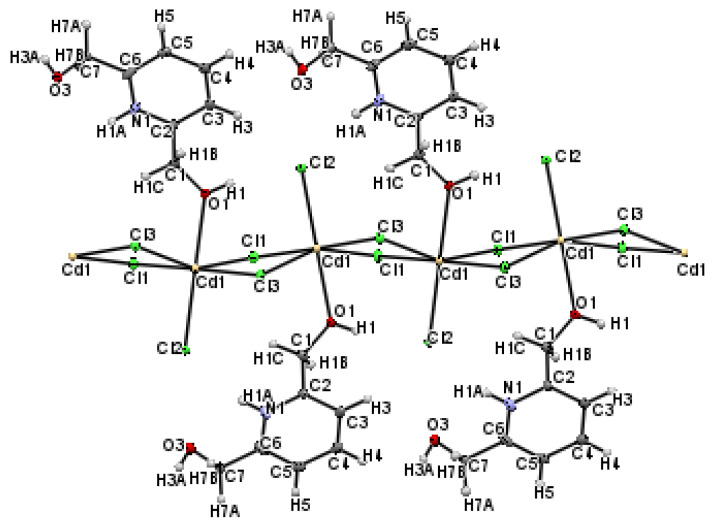
ORTEP plot of extended 1D polymeric chain showing the *µ*_2_-Cl bridges.

In the crystalline structure of (I), two neighboring cadmium atoms are connected by *µ*_2_-Cl bridges involving Cl1 and Cl3 atoms (Figure 4), leading to a 1D polymeric chain running along the *b*-axis.

As a result, five chlorine atoms and one oxygen atom surround each cadmium atom, constituting a deformed [CdCl_5_O]^−^ octahedron with the Cd–X bond length (X = Cl or O) range from 2.4214 (7) to 2.6372 (2) Å and the X–Cd–X range from 80.281 (17) to 174.545 (18)°. The distance between successive metal cadmium is 3.846 (0) Å and the Cd–Cd–Cd angle is 164.58(1)°, showing that the polymeric chains are approximately linear. These values are consistent with those found for other comparable halogenocadmate systems [47,48,49,50]. Equations (1) and (2) were used to obtain the average values of the cadmium octahedron distortion parameters [51]:

(1) $$ID(\mathrm{Cd}–X)=\sum_{i=1}^{n1}\frac{\left|Di-Dm\right|}{n1\;Dm}$$

(2) $$ID(X–\mathrm{Cd}–X)=\sum_{i=1}^{n2}\frac{\left|Ai-Am\right|}{n1\;Am}\;$$

where “*D*” represents the Cd–X bond length, “*A*” represents the X–Cd–X angle, “*m*” indicates the mean value, *n*1 = 6 (the octahedron’s bond number) and *n*2 = 12 (the octahedron’s cis angles). The distortion indices have values of *ID* (Cd–X) = 0.0199 and *ID* (X–Cd–X) = 0.0589.

**Figure 5:**
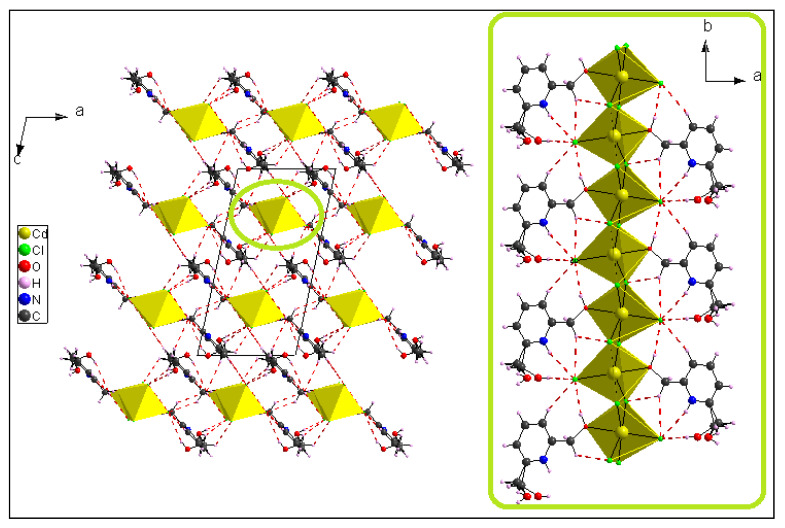
A view along the $\vec{b}$ axis on the crystal packing of compound (I) and of the polymeric chains running along the $\vec{b}$ direction.

From a crystallographic perspective, the 1D polymeric chains are localized on two-fold screw axes. Hence, the atomic arrangement is built by regularly applying the glide plane ***c*** and inversion center operations to the unit. These chains, located at (½, 0, ¼) and (½, 0, ¾), are linked to the organic molecule (C_7_H_10_NO_2_) via numerous H-bonds of type C–H**^...^**Cl, O–H**^...^**Cl and N–H**^...^**Cl to form columns running along the $\vec{b\;}$ direction. These columns are connected to one anotherby H-bonds of the types C–H**^...^**Cl and O–H**^...^**Cl to form a network in three dimensions (Table S2, included in the Supplementary Materials) (Figure 5).

**Figure 6:**
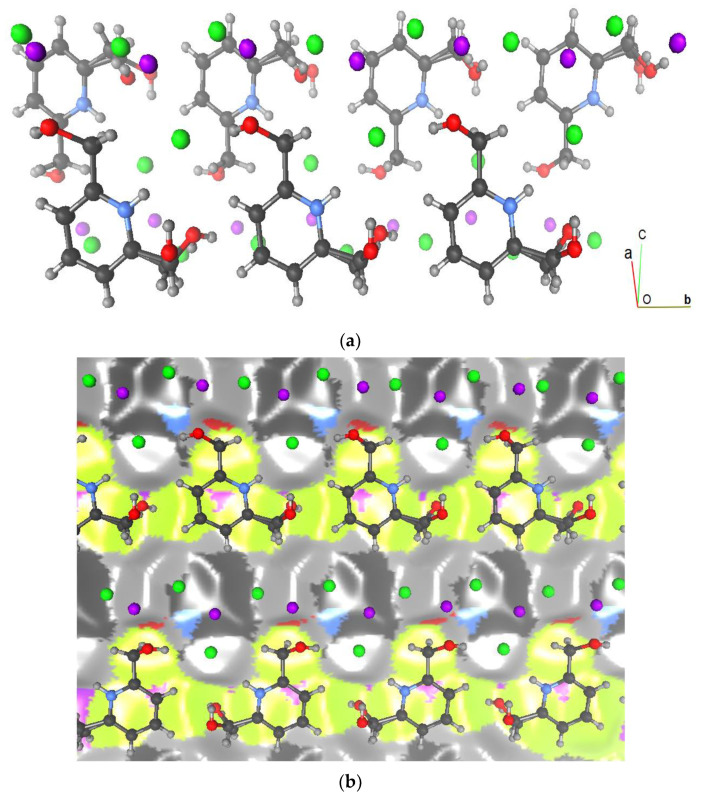
(**a**) Crystallographic autostereogram along the *a*-axis showing two layers of parallel aromatic cycles for compound (I). Hydrogen: grey; carbon: black; nitrogen: blue; oxygen: red; chlorine: green; cadmium: violet. (**b**) Crystallographic autostereogram of the Hirshfeld surface between two layers. The surface is colored according to the rear atom contributing most to the electron density.

All of the aromatic rings are arranged in a nearly parallel way and form layers oriented in planes (−102), which can be seen in Figure 6. A layer is made up of organic and inorganic moieties that alternate in the [−102] direction. The inorganic moieties appear to be located on both sides of the aromatic rings. Figure 6b highlights the large occurrence of the H…Cl^−^ and C…Cl^−^ contacts as the chlorine anions are located above the pyridinium cycle.

**Figure 7:**
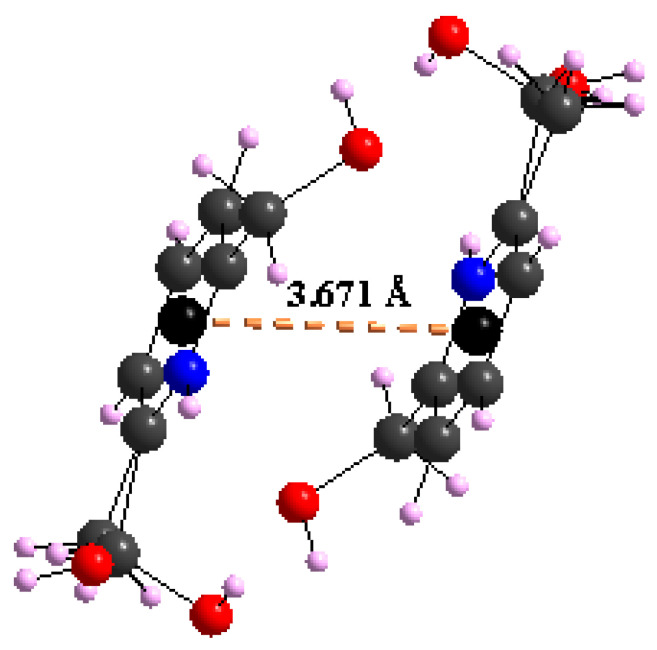
Aromatic stacking π…π within compound (I).

Furthermore, the crystal packing is strengthened by the intermolecular parallel displaced stacking interaction between adjacent aromatic rings, with a centroid–centroid distance equal to 3.671(4) Å (Figure 7). Concerning the ligand, it presents a regular configuration where the C=C and C=N vary from 1.373 (1) to 1.389 (1) Å and 1.349 (1) to 1.352 (1) Å, respectively (Table S1). Within the [C_7_H_10_NO_2_]^+^ ligand, the uncoordinated methoxy group (–CH_2_–OH) exhibits a disorder over two sets of atomic sites with 0.523 (2)/0.477 (2) occupancies, whereas the coordinated hydroxyl (–OH) group deviates slightly from the 2,6-dimethanolpyridinium plane with 59.21 (6)°.

#### 3.2.2. Crystal Structure of [(C_7_H_9_NO_2_)CuCl_2_] (II)

**Figure 8:**
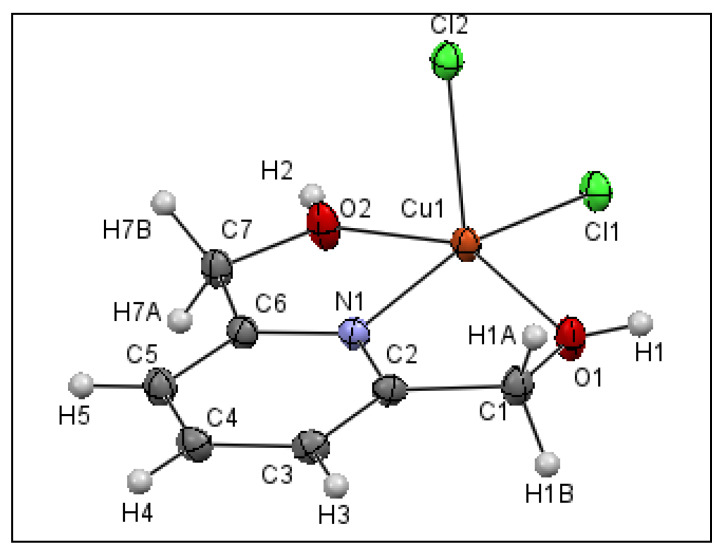
ORTEP plot showing at the 50% probability level the anisotropic displacement parameter of compound (II).

According to the crystallographic study, the title compound crystallizes in the triclinic space group *P*$\bar{1}$. Figure 8 displays the asymmetric unit, revealing that compound (II) is a mononuclear copper (II) complex and the central metal exhibits a penta-coordinated environment.

The tridentate bridging ligand is coordinated to Cu (II) with nitrogen N(1) and its two oxygen atoms O(2) and O(1). The O(1), O(2), N(1) and Cl(1) atoms define the equatorial plane of a deformed square pyramid, and the chlorine atom Cl(2) occupies the axial position, with a bond length of Cu–Cl(2) = 2.5062 (4) Å, which is longer than the equatorial bonds, and the bond angles between the axial atom Cl(2), the central atom Cu and the equatorial atoms are Cl2–Cu–Cl1 = 100.192(15)°, Cl2–Cu–O1 = 102.13(4)°, Cl2–Cu–O2 = 98.03(4)° and Cl2–Cu–N = 97.31(4)°. The dihedral angle between the plans formed by (C6, N1, Cu, O2, C7) and (Cu, O1, C1, C2, N1) is 12.350 (35)°. Furthermore, the pyridyl N atoms are considered strongly coordinated to the metal with a bond length of Cu–N of 1.9356(12) Å, while the oxygen and chlorine atoms of the equatorial plane form weaker connections with the central metal with a distance ranging from 2.0305(12) to 2.2228(4) Å (Table S1). All of these values are consistent with those found for other comparable systems [52,53,54,55,56]. To differentiate between trigonal bipyramidal and square pyramidal coordination centers, Addison et al. suggested the “*τ*_5_” parameter using the following Equation [57]:

(3) $$\tau_{5}=\frac{\beta -\alpha }{60}$$

where *β* > *α* represents the coordination center’s two largest valence angles. If τ_5_ is near 0, the geometry is similar to that of a square pyramid, whereas when τ_5_ is near 1, the geometry is similar to that of a trigonal bipyramid. In this case, τ_5_ is equal to 0.176. This value confirms the presence of a deformed square pyramidal shape for complex (II), where the Cu (II) ion is raised by 0.3751 Å from the plane formed by (O1, C1, C7, O2). It is worth mentioning that the [(C_7_H_9_NO_2_)CuCl_2_] complex (complex (II)) is solvatomorphic with [(C_7_H_9_NO_2_)CuCl_2_]·H_2_O and isostructural with [(C_7_H_9_NO_2_)ZnCl_2_] [58], where the central metal is five-coordinated, giving a slightly distorted square-pyramidal geometry (τ_5_ = 0.015).

**Figure 9:**
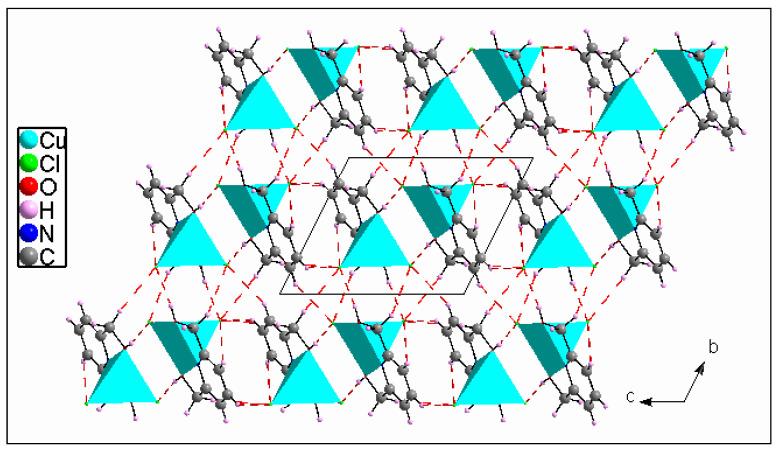
A view along the *a*-axis of the crystal packing. Red dashed lines represent the H-bonds of compound (II).

**Figure 10:**
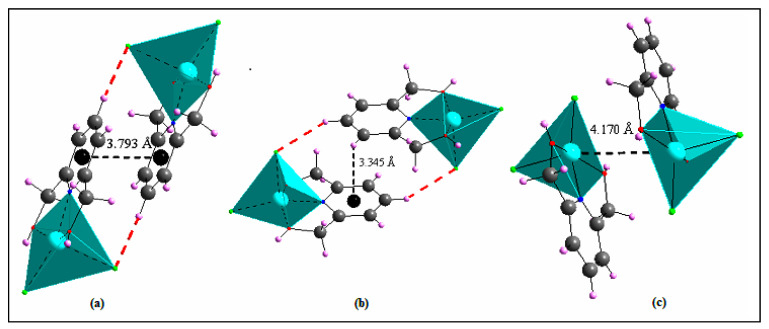
Aromatic stacking (**a**), C–H…π (**b**) and Cu…Cu (**c**) interactions within compound (II).

The crystal structure is built by neighboring complex cations that are connected to each other via Cl1...H2–O2, Cl1...H1B–C1, Cl2...H3–C3 and Cl2...H7B–C7 H-bonds to create a layer extending parallel to the *ac*-plane at *y* = ½ (Table S2). These layers are interconnected via Cl1...H5–C5, Cl1...H1A–C1, Cl2...H1–O1 andCl2...H7A–C7 hydrogen bonds giving rise to a 3D metal–organic framework (Figure 9). Other intermolecular interactions, such as C–H...π (about 3.345 Å, Figure 10a), aromatic stacking (about 3.793 Å, Figure 10b) and Cu...Cu (about 4.170 Å, Figure 10c), also contribute to the crystal’s cohesiveness.

The most noticeable difference between the two compounds is in the arrangement of the 2,6-dimethanol pyridine ligand around the central metal. Indeed, the coordination in complex (I) is octahedral around the core metal. The apical position is occupied by the oxygen O1, whereas in complex (II), the plane of square pyramidal geometry surrounding the core metal is made up of all three donors (O, N and O’) of the ligand. Indeed, the electrical interactions between the metal and the ligand cause spatial configuration differences as well as variation in coordination distances, such as the distance between O1 and Cd (2.4214 (7) Å), which is longer than O–Cu (~2.02 Å). The bond lengths and angles in both compounds of the 2,6-dimethanol pyridine ligand are shown in Table S1 and are comparable to other compounds containing the same ligand [59,60,61,62,63].

### 3.3. Hirshfeld Surface Analysis

**Figure 11:**
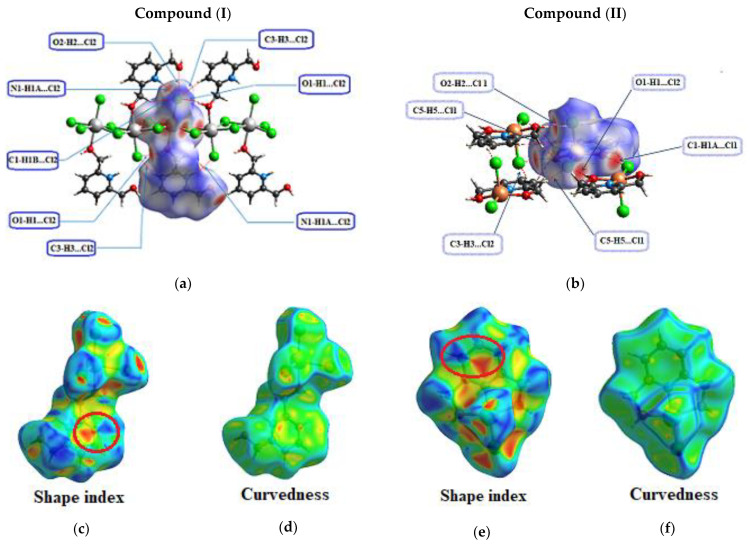
View of the 3D Hirshfeld surface plotted over *d*_norm_ (**a**,**b**), around the asymmetric unit for both compounds. Shape index (**c**,**e**) and curvedness (**d**,**f**), respectively for compound (I) and compound (II).

To explore the nature of the intermolecular interactions, the Hirshfeld surface analysis was utilized. The normalized contact distance *d*_norm_ has been shown to be an incredibly effective tool for identifying zones of close contact [64,65]. Figure 11 depict the *d*_norm_ maps of the compounds (I) and (II) (Figure 11a,b), respectively, which indicate the relative placement of adjacent atoms from different interacting molecules. Deep-red spots around the asymmetric unit may be recognized for compound (I) as N–H^...^Cl, O–H^...^Cl, C–H^...^Cl and C–H^...^O contacts, and O–H^...^Cl and C–H^...^Cl contacts for compound (II). The presence of stacking interactions is verified by the set pattern of neighboring blue and red triangles on both compounds’ shape index surfaces (Figure 11c,e), as well as a considerably large and flat green region on the same side of the associated curvedness surfaces. A smaller flat-green zone on compound (II)’s curvedness surface allows us to assess the presence of stacking with a minor overlapping of the neighboring molecules (Figure 11d,f).

The statistics on intermolecular contacts are shown in Table 2. The obtained contact percentages are used to compute the enrichment ratio *E*_XY_ of the major intermolecular interactions [66]. These data aid in determining the high and low propensities of a couple of chemical elements (X, Y) to make contacts in the crystal packing. In fact, a couple of elements (X, Y) with a ratio of enrichment higher than the unit have a strong potential to create contacts in a crystal. If the *E*_XY_ value is less than unity, the pair (X, Y) tends to avoid contact with each other.

For both structures, the Cl^−^...H hydrogen bonds contribute the most to the total Hirshfeld surface (35.4% and 34.9% for complex(I) and complex (II), respectively). Both strong Cl^−^… H-O/H-Nand weak Cl^−^…H-C hydrogen bonds are moderately overrepresented (*E* is between 1.66 and 2.31). Complex (I)’s structure is ensured by eight C–H...Cl^−^ bond types, three O–H...Cl^−^ bond types, and one N–H...Cl^−^ H-bond type. Complex (II), on the other hand, has six C–H...Cl^−^ and two O–H...Cl^−^ H-bond types (Table S2). The H...Cl^−^ contact emerges as an extremely sharp spike in the 2D fingerprint plots for (I) and (II), which is characteristic of strong interactions, but there are also weaker contacts at longer distances. The plots showing the results of this investigation are given in the Supplementary Materials (Figure S1).

Table 2 shows that the Cd(II) cation is almost completely surrounded by chloride anions, with enrichment of *E*_Cd,Cl_ = 2.28. In complex (II), the Cu(II) cation is surrounded by two chloride anions and by two hydroxyl oxygen atoms. Both of these contacts are over-represented (1.4 and 4.3, respectively).

Other weak intermolecular interactions were detected using the Hirshfeld surface analysis, albeit their percentage contributions are lower (Table 2). Indeed, there are many different contact types in the crystal packing. The hydrophobic interactions involving only H-C and C atoms represent 8% and 17.8%, respectively, in compounds (I) and (II), and therefore Vander Waals interactions are more important in the second crystal structure. The C...H-C very weak hydrogen bonds are underrepresented in compound (I) with *E*_C…H-C_ = 0.49, whereas for compound (II) the enrichment is close to the unit (*E*_C…H-C_ = 1.071). The C...C contacts are quite enriched for both compounds: *E*_CC_ = 3.16 for (I) and 2.62 for (II). In both crystals, all of the aromatic cycles are parallel and are involved in parallel displaced stacking. The Cl^−^...C contacts represent up to 8.3% and 6.3% for compounds (I) and (II), respectively, due to the presence of chloride anions on both sides of the aromatic ring. Their enrichment is not far from the unit.

### 3.4. Vibrational Study

**Figure 12:**
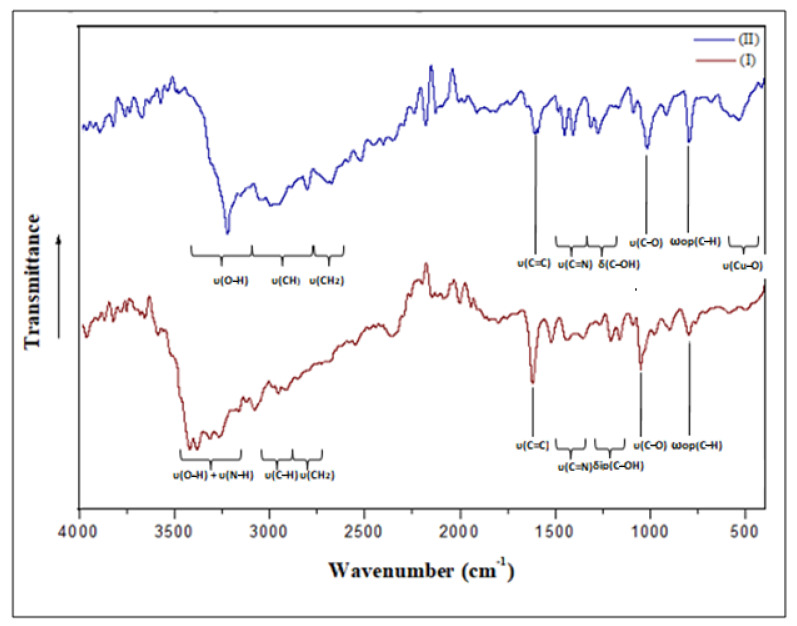
The infrared spectra, recorded at room temperature, of complex (I) and complex (II).

Using infrared absorption spectroscopy, we were able to gather information on the crystal structure and better understand the effects of coordination on vibrational behavior. For this reason, the IR spectra of (I) and (II) recorded at room temperature are given in Figure 12, showing great analogies in agreement with their structural similarities (Table S3, included in the Supplementary Materials). All frequency allocations were determined by comparing the measured frequencies to the ligand spectrum accessible in bibliographic sources, based on other compounds associated with the same organic cations [67,68].

At high frequencies, the bands observed in the IR spectrum of complex (I) between 3425 and 3258 cm^−1^ are ascribed to υ(OH) and υ(NH). However, upon complexation, as in complex (II), this band becomes broader and shifts towards lower frequencies, appearing as a broad band centered at 3221 cm^−1^, indicating that the coordination of Cu(II) occurs via the two hydroxyl oxygen atoms and nitrogen atom. The absence of an N-H band in the spectrum of compound (II) indicates that its nitrogen atom is not protonated as in compound I. In fact, the presence of an extensive hydrogen bonding system in the complexes, as well as the nature of the interaction between the ligand’s oxygen atom and the ion (Cd(II) or Cu(II)), can be ascribed to band enlargement in this region. The two observed bands at 3150 cm^−1^ and 3078 cm^−1^ for complex (I) and the board band in the 3030–2924 cm^−1^ range for complex (II) are attributed to the stretching vibrations of the CH groups engaged in H-bonds (Table S2). As for the band appearing at 2968 cm^−1^ and the shoulder band at 2913 cm^−1^ in the IR spectrum of (I), as well as the sharp band at 2810 cm^−1^ in the IR spectrum of complex (II), they are assigned to υ(CH_2_). With reference to the free organic cation spectrum [35], the band due to the *υ*(C–O) vibration appearing at 1084 cm^−1^ is shifted to lower wavenumbers—to 1055 cm^−1^ for (I) and to 1014 cm^−1^ for (II). Moreover, the broad bands appearing in the IR spectrum of (II) in the range of 408–524 cm^−1^ and absent in the IR spectrum of (I) are attributed to the Cu–O and Cu–N vibrations within complex (II). In fact, the shorter the M–O bond, the stronger and broader the band [69]. In this case, the band of compound (II) is larger than that of complex (I)(Cu–O distance is around 2.03Å and Cd–O = 2.4214 Å). All of these results agree well with the XRD results.

### 3.5. Optical Study

**Figure 13:**
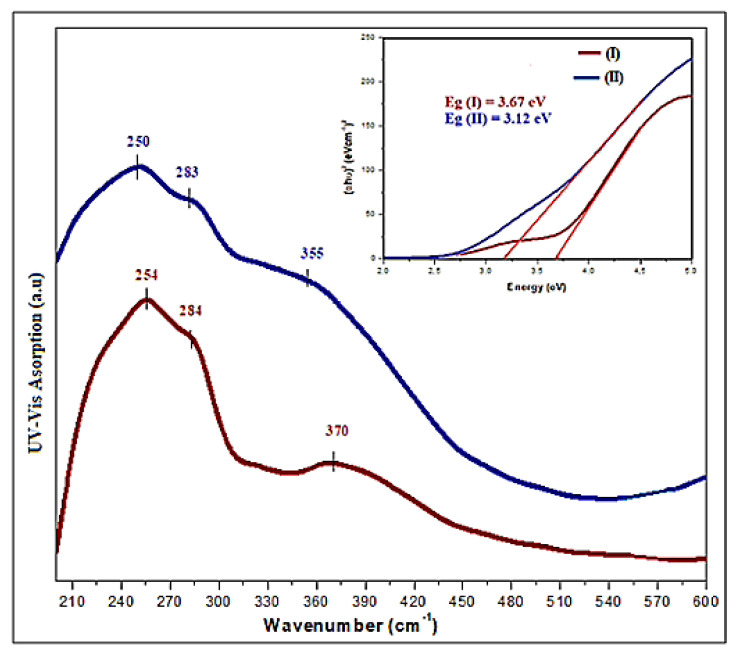
The solid-state UV–visible spectrum at room temperature. Tauc’splot giving the energy gap for complexes (I) and (II).

The solid-state UV absorption spectra of complexes (I) and (II) at room temperature are shown in Figure 13. The observed absorption bands at 254 nm for complex (I) and 250 nm for complex (II) are attributed to the organic part’s π→π* transitions [70]. The bands observed around 284 and 370 nm for compound (I) are ascribed to the absorption of the maximum energetic level in the conduction band, indicating the gap in the material. In fact, the band at 284 nm is mainly caused by the absorption between O (2s or 2p) and Cd (5s) (band to band), whereas the band at 370 nm is attributed to the excitation of free electron–hole pairs due to the absorption between Cl (3p) and Cd (5s). However, for complex (II), the band at 283 nm is described as the transition between the valence band Cl (3p) and the conduction band Cu (4s), whereas the band observed at 331 nm is caused by the photo-induced exciton generated by the transition from the top of the valence band made by Cl (3p) to the bottom of the Cu (3d). It is worth noting that due to the presence of covalently bonded oxygen and copper, the bands in the UV-visible spectrum of complex (II) are wider [71].

The optical band gap energy is located between the valance and the conduction bands. Tauc’s model was used to measure the optical band gap energy [72] in order to estimate the reactivity and stability of these compounds, by the use of the equation:

(4) $${\left(\alpha h\upsilon \right)}^{1/2}=A\;\left(h\upsilon -E_{g}\right)\;\;\;\;\;$$

where *A* is a constant, *hυ* represents the energy of an incident photo and *E*_g_ is the crystal’s bandgap energy. We may obtainthe indirect *E*_g_ by the projection of the energy *hυ* intercept using Equation (3). Based on the curve in Figure 11, the optical gap energy for complex (I) is 3.67 eV and 3.12 eV for complex (II). According to the literature, photovoltaic materials for domestic solar radiation in solar-cell applications have properties that are quite similar to those of semiconductor materials [73].

### 3.6. Thermal Analysis

TG-DTA is a technique frequently used to characterize complexes from a thermal point of view based on their physical and chemical properties. The thermal profiles were obtained by performing simultaneous TG-DTA in an argon atmosphere between 280 and 700 K.

**Figure 14:**
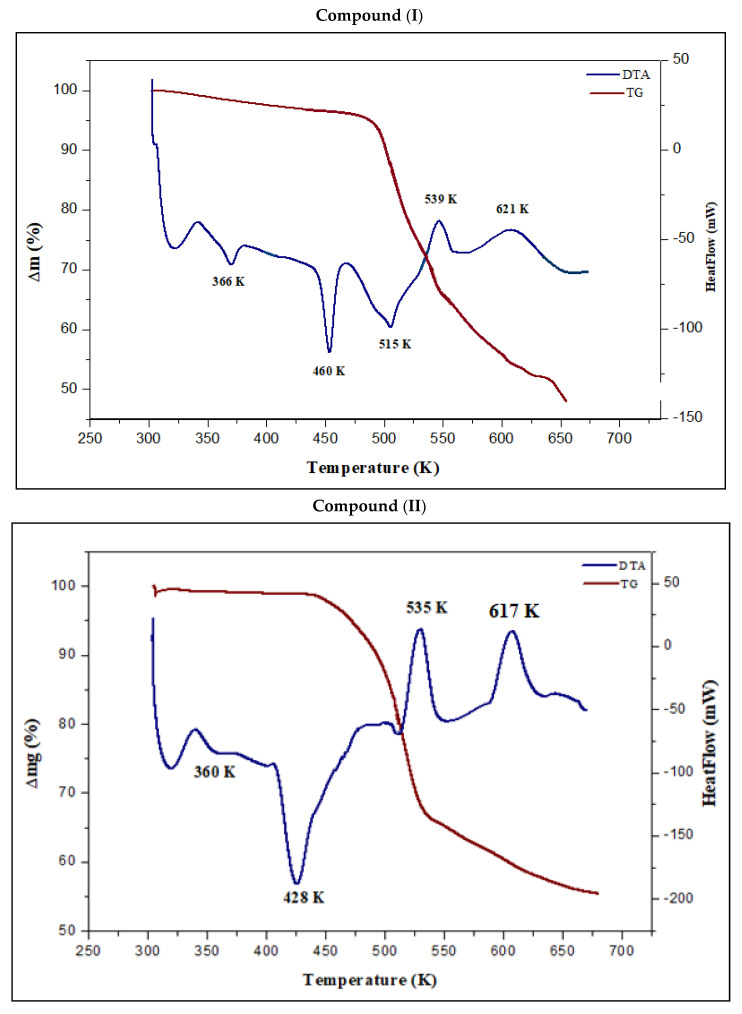
TG–DTA curves for compound (I) and compound (II).

Figure 14 shows the results of the thermal study of complexes (I) and (II). The first peak in the DTA complex (I) occurs at 366 K (ΔH = 149.151 J·g^−1^) and can be attributed to the loss of the uncoordinated (–OH) group with a weight loss of 4.46% (calc. 4.74%). After the dehydroxylation, the compound is stable up to 435 K before it starts to melt and decompose. The melting of complex (I) is responsible for the second endothermic peak at 460 K (Δ*H* = 322.185 J·g^−1^). This result was proven by the use of a Koffler heating bank. The decomposition of this complex starts at 462 K (onset). The second weight loss, which occurs between 563–558 K, equates to a total weight reduction of around 25.73%, which is quite near to the calculated value of 25.38% for the decomposition of the pyridinium ring, as can be seen in the DTA curve as a peak at 515 K (Δ*H* = 595.768 J·g^−1^) and a peak at 539 K (Δ*H* = −222.38 J·g^−1^). The mass loss occurs at 621 K (Δ*H* = −609.725 J·g^−1^), comprising 19.79% of the total mass (calc 19.75%), and is due to the emission of chloride gas. The end of the experience was reached when cadmium oxide was formed and the entire decomposition was completed, which caused a black carbon residue to form and the emission of a nauseating gas. For complex (II), the first peak in the DTA curve occurs at 360 K and can be attributed to the phase transition without any mass loss observed on the TG curve (Δ*H* = 82.433 J·g^−1^). The following transformation observed at 428 K (onset at 409 K) corresponds to the melting (Δ*H* = 474.815 J·g^−1^). These results were further confirmed by using the Koffler heating bank. The decomposition step for this complex is accomplished in two main steps. The initial weight loss of 34.26% (calc. 34%) occurs at 535 K (onset 517 K), which is accompanied by an exothermic effect associated with the elimination of the organic ligand (Δ*H* = −658.076 J·g^−1^). Above 597 K, the second mass loss of about 35.8% (calc. 36.1%) complies with the release of chloride gas. Metal oxide is formed as a result of further heating. Hence, the thermal investigation aimed at determining how the structural characteristics of metal ions affect the thermal behavior.

## 4. Conclusions

In the present contribution, we tried to explore the impact of cadmium and copper on ligand flexibility. Both compounds’ crystal packing is maintained by several hydrogen bonds and ionic interactions. The crystallographic analysis revealed that for complex (II), instead of the octahedral geometry, the tridentate ligand induces the formation of a square pyramidal environment, which is normally preferred by the d^10^ configuration as for compound (I). XRPD patterns from the experimental and the single-crystal simulations showed that the phases of the two complexes (I) and (II) were indeed pure, and this was validated by the comparison of the two sets of XRPD patterns. The intensities of the experimental and simulated XRPD patterns fluctuate because the preferred orientation of the powder sample varies in the experimental XRPD. SEM was used to establish surface homogeneity, and EDX was used to confirm the composition. The Hirshfeld surface analysis showed that, for both compounds, the intermolecular interactions H…Cl account for an important percentage of the overall surface area, followed by H…H contacts. It also indicated that metal coordination and hydrogen bonding are the primary driving forces in the crystal packing creation. The aromatic cycles are all parallel and arranged in layers for both compounds. The UV-visible spectral analysis revealed that the synthesized materials can be used as semiconductor materials, whereas infrared spectroscopy showed that compounds (I) and (II) exhibit structural deformation when compared to 2,6-dimethanolpridine’s IR spectra, indicating the complexation in two different ways. The thermal stability of the materials was evaluated using TG-DTA methods.

## Figures and Tables

**Table 1:** Details on crystal data and structure refinement.

	Compound (I)	Compound (II)
Crystal data		
Chemical formula	C_7_H_10_CdCl_3_NO_2_	C_7_H_9_Cl_2_CuNO_2_
Mr(g.mol^−1^)	358.91	273.59
Crystal system Space group	Monoclinic *P*2_1_/c	Triclinic *P* $\bar{1}$
Temperature (K)	100	100
a, b, c (Å)	8.65880(10), 7.62320(10), 16.8690 (2)	7.0814(5), 7.9041(6), 9.6875(7)
α (°)	90	113.196(2)
β (°)	102.2540(10)	104.142(2)
γ(°)	90	98.578(2)
Volume (Å^3^)	1088.12 (2)	464.61 (6)
Z	4	2
Radiation type	Mo Kα	Mo Kα
Absorption μ (mm^−1^)	2.715	2.89
Crystal size (mm)	0.159 × 0.112 × 0.109	0.23 × 0.20 × 0.15
Data collection		
Diffractometer	SuperNova, Atlas CCD	D8 Venture, PIII C14
Absorption correction	Multi-scan	Multi-scan
Tmin, max	0.760, 0.950	0.595, 0.747
No. of measured, independent and observed (I > 2σ(I))	76,249, 8978, 7772	21,702, 4496, 3914
R_int_	0.0324	0.040
(sin θ/λ)max(Å^−1^)	0.997	0.834
Refinement		
R[F^2^ > 2σ(F^2^)], wR(F^2^), S	0.0204, 0.0484, 1.156	0.032, 0.08, 1.07
No. of reflections	8978	4496
No. of parameters	151	120
No. of restraints	8	0
Δρmax, Δρmin(eÅ^−3^)	1.33, −1.18	0.72, −1.25
CCDC No	2,104,624	2,104,625

**Table 2:** Hirshfeld contact surface and enrichment ratios.

Compound (I)							
Atom	Cd	Cl	O	H-O/H-N	N	H-C	C
Surface%	11.8	35.5	8.3	9.8	0.9	21.6	12.2
Cd	0.0	**24.8**	3.5	0.3	0.0	1.1	0.5
Cl		0.8	1.8	**11.7**	0.7	**23.7**	**8.3**
O			0.3	2.5	0.3	4.3	2.0
H-O/H-N		Actual		0.0	0.0	3.8	1.0
N		Contacts	(**%**)		0.0	0.1	0.6
H-C						2.1	2.1
C							3.8
Cd	0.01	**2.28**	**1.49**	0.09	/	0.19	0.14
Cl		0.06	0.32	**1.67**	/	**1.66**	1.04
O			/	**1.73**	/	**1.46**	1.21
H-O/H-N					/	1.01	0.46
H-C		Enrichment			/	0.55	0.49
C					/		**3.16**
**Compound (II)**							
Atom	Cu	Cl	O	N	H-O/H-N	Hc	C
Surface%	13.2	28.0	7.0	2.9	7.2	26.9	14.7
Cu	1.3	**14.7**	**10.1**	4.6	1.7	1.4	2.3
Cl		0.2	0.0	0.1	**8.5**	**26.4**	6.3
O			0.2	0.0	0.0	1.0	0.5
N		Actual		0.0	0.0	0.0	0.2
H-O/H-N		Contacts	**%**		0.0	1.7	1.2
H-C						6.7	6.9
C							4.2
Cu	0.40	**1.40**	**4.30**	**4.78**	0.69	0.15	0.45
Cl		0.02	0.00	0.07	**2.31**	**1.85**	0.87
O			/	/	/	0.33	0.33
N				/	/	0.00	/
H					/	0.50	0.71
H-C		Enrichment				1.04	1.07
C							**2.62**

/: Enrichment ratios for random contacts lower than 0.9% are not shown because they are not meaningful.

## Data Availability

Not applicable.

## Supplementary Materials

The following supporting information can be downloaded at: https://www.mdpi.com/article/10.3390/ma15051624/s1, Figure S1: The 2D fingerprint plots of the main contacts around the ligand for both compounds; Table S1: Bond lengths and angles for compound (I) and compound (II); Table S2: Non-covalent interactions in the crystal structure of compound (I) and (II). Table S3: Comparison of the IR frequencies of compound (I) and (II).
