# Supplementary material for: The Coordination Behavior of Two New Complexes, [(C7H10NO2)CdCl3]n(I) and [(C7H9NO2)CuCl2] (II), Based on 2,6-Dimethanolpyridine; Elaboration of the Structure and Hirshfeld Surface, Optical, Spectroscopic and Thermal Analysis
Source: Materials (Basel). 2022 Feb 22;15(5):1624. doi: 10.3390/ma15051624 (PMC8911489; doi:10.3390/ma15051624)
Supplement: Supplementary file 1 [file materials-15-01624-s001.zip › materials-1581644-supplementary.pdf]

materials

MDPI

Compound (I)

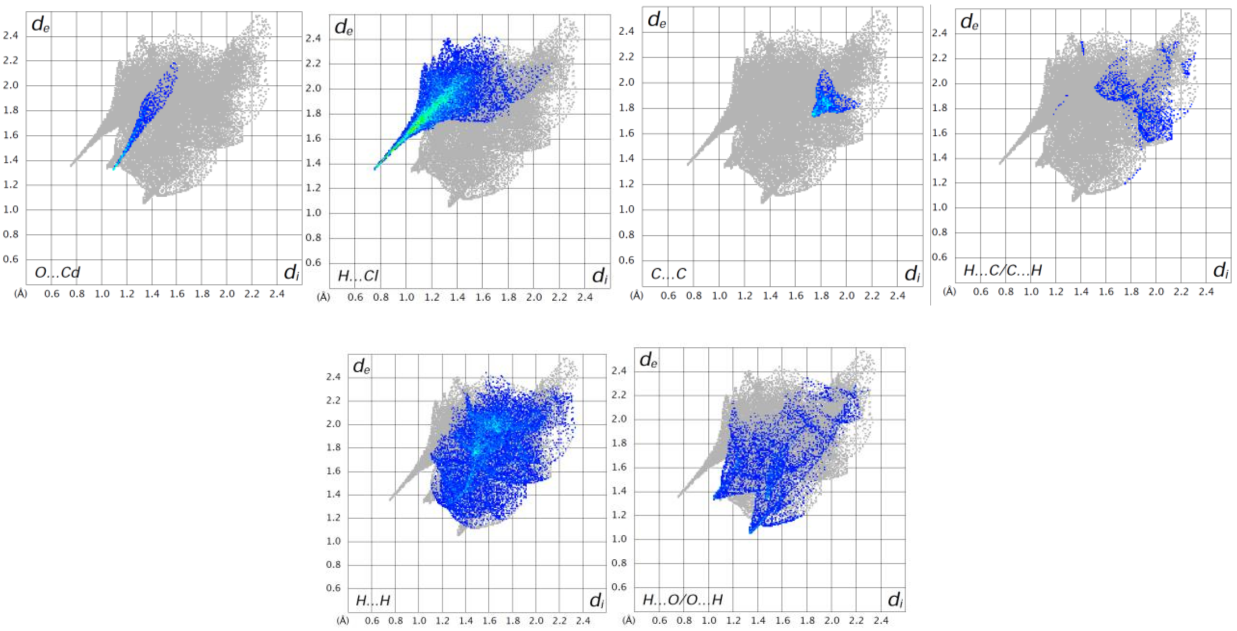

Compound (II)

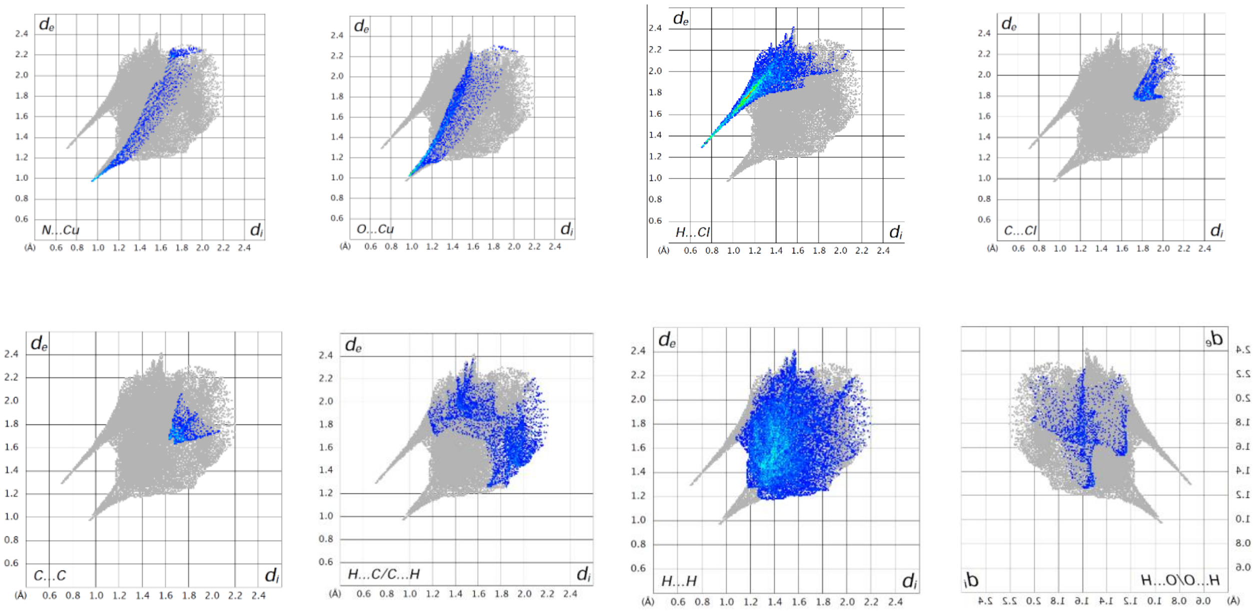

Figure S1. The 2D fingerprint plots of the maincontacts around the ligand for both compounds.

Table S1. Bond lengths and angles for compound (I) and compound (II).

Compound (I)			
**Bond**	***d*** **(Å)**	**Bond**	***d*** **(Å)**
Cd1—Cl3[i]	2.6372 (2)	N1—C6	1.3517 (13)
Cd1—Cl3	2.6153 (2)	C2—C1	1.5029 (12)
Cd1—Cl1	2.5948 (2)	C2—C3	1.3796 (13)
Cd1—Cl1[ii]	2.6047 (2)	C5—C4	1.3876 (14)
Cd1—Cl2	2.5777 (2)	C5—C6	1.3733 (15)
Cd1—O1	2.4214 (7)	C4—C3	1.3891 (13)
O1—C1	1.4200 (11)	C6—C7	1.455 (4)
N1—C2	1.3488 (11)	O3—C7	1.415 (4)
C8—O2	1.426 (4)	C6—C8	1.571 (4)
**Angles (°)**	***ω*** **(°)**	**Angles (°)**	***ω*** **(°)**
Cl3—Cd1—Cl3[1]	167.972(4)	C6—C5—C4	119.17(9)
Cl1—Cd1—Cl3[1]	84.090(7)	C5—C4—C3	120.37(9)
Cl1[2]—Cd1—Cl3[1]	89.977(6)	O1—C1—C2	112.56(7)
Cl1[2]—Cd1—Cl3	84.331(7)	N1—C6—C5	118.84(8)
Cl1—Cd1—Cl3	99.587(7)	N1—C6—C8	111.33(17)
Cl1—Cd1—Cl1[2]	168.469(3)	N1—C6—C7	121.45(15)
Cl2—Cd1—Cl3[1]	95.087(7)	C5—C6—C8	129.54(18)
Cl2—Cd1—Cl3	96.125(7)	C5—C6—C7	119.70(15)
Cl2—Cd1—Cl1[2]	97.128(7)	C7—C6—C8	11.94(18)
Cl2—Cd1—Cl1	93.248(7)	C2—C3—C4	119.38(9)
O1—Cd1—Cl3	80.281(17)	O2—C8—C6	113.6(3)
O1—Cd1—Cl3[1]	88.834(17)	O3—C7—C6	107.2(3)
O1—Cd1—Cl1	83.368(17)	C1—O1—Cd1	120.50(5)
O1—Cd1—Cl1[2]	86.643(17)	C2—N1—C6	123.89(8)
O1—Cd1—Cl2	174.545(18)	N1—C2—C1	117.01(8)
Cd1—Cl3—Cd1[2]	94.156(6)	N1—C2—C3	118.35(8)
Cd1—Cl1—Cd1[1]	95.420(7)	C3—C2—C1	124.60(8)
(i) –x+1, y-1/2, -z+1/2; (ii) –x+1, y+1/2, -z+1/2.			
**Compound (II)**			
**Bond (Å)**	***d*** **(Å)**	**Bond**	***d*** **(Å)**
Cu1—Cl1	2.2228 (4)	N1—C6	1.3437 (19)
Cu1—Cl2	2.5062 (4)	N1—C2	1.3438 (18)
Cu1—O1	2.0120 (12)	C6—C7	1.505 (2)
Cu1—O2	2.0305 (12)	C6—C5	1.383 (2)
Cu1—N1	1.9356 (12)	C4—C3	1.394 (2)
O1—C1	1.4379 (19)	C4—C5	1.394 (2)
O2—C7	1.4309 (19)	C1—C2	1.504 (2)
C3—C2	1.389 (2)		
**Angles (°)**	***ω*** **(°)**	**Angles (°)**	***ω*** **(°)**
C3—C4—C5	119.94 (13)	O2—Cu1—Cl1	97.66 (3)
O1—C1—C2	107.41 (11)	O2—Cu1—Cl2	98.03 (4)
C2—C3—C4	118.37 (13)	N1—Cu1—Cl1	162.48 (4)
O2—C7—C6	108.03 (12)	N1—Cu1—Cl2	97.31 (4)
C6—C5—C4	118.76 (14)	N1—Cu1—O1	79.11 (5)
N1—C2—C1	114.82 (12)	N1—Cu1—O2	79.09 (5)
N1—C2—C3	120.84 (13)	C1—O1—Cu1	115.24 (9)
C3—C2—C1	124.35 (13)	C7—O2—Cu1	117.01 (9)

Cl1—Cu1—Cl2	100.192 (15)	C6—N1—Cu1	119.75 (10)
O1—Cu1—Cl1	97.81 (4)	N1—C6—C7	115.27 (12)
O1—Cu1—Cl2	102.13 (4)	N1—C6—C5	120.72 (13)
O1—Cu1—O2	151.90 (5)	C6—N1—C2	121.35 (12)
C5—C6—C7	124.01 (13)	C2—N1—Cu1	118.77 (10)

Table S2. Non-covalent interactions in the crystal structure of compound (I) and (II).

**Compound (I)**				
**D–H...A**	**d(D–H) (Å)**	**d(H–A) (Å)**	**d(D–A) (Å)**	**D–H...A (°)**
O1–H1...Cl2[1]	0.858 (8)	2.289 (8)	3.1294 (7)	166.5 (13)
O2–H2...Cl2[2]	0.84	2.84	3.6676 (19)	163.2
O3–H3A...Cl3[6]	0.84	2.26	3.1002 (14)	176.0
C1–H1B...Cl2[4]	0.99	2.75	3.5511 (11)	138.3
C1–H1C...Cl3[2]	0.99	2.89	3.4279 (9)	114.8
C1–H1C...Cl2[2]	0.99	2.76	3.6778 (10)	154.6
C3–H3...Cl2[1]	0.95	2.84	3.7443 (9)	159.0
C5–H5...Cl3[3]	0.95	2.80	3.6630 (9)	150.8
C7–H7A...Cl1[4]	0.99	2.87	3.453 (4)	118.8
C7–H7B...Cl2[7]	0.99	2.94	3.921 (5)	170.4
C8–H8A...Cl3[6]	0.99	2.92	3.832 (5)	153.5
C8–H8B...Cl3[3]	0.99	2.92	3.827 (4)	152.5
C3–H3...O3[5]	0.95	2.46	3.0752 (16)	122.1
N1–H1A...Cl2[2]	0.88	2.53	3.3854 (9)	165.8
(1) 1-*x*, ½+*y*, ½-*z*; (2) 1-*x*, - ½+*y*, ½-*z*; (3) 2-*x*, 1-*y*, 1-*z*; (4)1+*x*, *y*,*z*; (5) *x*, 1+*y*,*z*; (6) 1+*x*, -1+*y*, *z*; (7) 1+*x*, ½-*y*, ½+*z*.				
**Compound (II)**				
**D–H...A**	***d*(D–H) (Å)**	***d*(H–A) (Å)**	***d*(D–A) (Å)**	**D–H...A (°)**
O1–H1...Cl2[1]	0.84	2.16	2.9950 (12)	170.7
O2–H2...Cl2[2]	0.84	2.23	3.0587 (12)	168.4
C1–H1A...Cl1[1]	0.99	2.76	3.6594 (16)	151.3
C1–H1B...Cl1[3]	0.99	2.89	3.8666 (16)	168.2
C3–H3...Cl2[3]	0.95	2.86	3.7054 (15)	148.6
C5–H5...Cl1[6]	0.95	2.90	3.6505 (16)	136.9
C7–H7A...Cl2[4]	0.99	2.98	3.9522 (16)	168.7
C7–H7B...Cl2[5]	0.99	2.98	3.8659 (16)	149.4
(1) 1-*x*, -*y*,1-*z*; (2) 2-*x*, 1-*y*, 1-*z*; (3) -1+*x*, *y*, *z*; (4) x, 1+*y*, *z*; (5) 2-*x*, 1-*y*, 2-*z*; (6) *x*, 1+*y*, 1+*z*.				

Table S3. Comparison of the IR frequencies of compound (I) and (II).

Assignments	Compound (I)	Compound (II)
$\upsilon_{(OH)}$	3424<br>3380	3221
$\upsilon_{(NH)}$	3320<br>3259	-
$\upsilon_{(CH)}$	3155<br>3078	3030<br>2924
$\upsilon_{(CH2)}$	2968<br>2913	2798<br>2687
$\upsilon_{(C=C)}$	1627	1599
$\upsilon_{(C=N)}$	1429<br>1395	1519
$\delta_{in(C-OH)}$	1207<br>1157	1269
$\upsilon_{(C-O)}$	1055	1019
$\omega_{op(CH)}$	795	797
